# The FSH-inhibin axis in prader-willi syndrome: heterogeneity of gonadal dysfunction

**DOI:** 10.1186/1477-7827-10-39

**Published:** 2012-05-06

**Authors:** Varda Gross-Tsur, Harry J Hirsch, Fortu Benarroch, Talia Eldar-Geva

**Affiliations:** 1 Multidisciplinary Prader-Willi Syndrome Clinic, Shaare Zedek Medical Center, Jerusalem, Israel; 2 Neuropediatric Unit, Department of Pediatrics, Shaare Zedek Medical Center, Jerusalem, Israel; 3 The Hebrew University Faculty of Medicine, Jerusalem, Israel; 4 Child and Adolescent Psychiatry, Hadassah Mount Scopus Hospital, Jerusalem, Israel; 5 Reproductive Endocrinology and Genetics Unit, Department of Obstetrics and Gynecology, Shaare Zedek Medical Center, Jerusalem, Israel

## Abstract

**Background:**

We characterized the spectrum and etiology of hypogonadism in a cohort of Prader-Willi syndrome (PWS) adolescents and adults.

**Methods:**

Reproductive hormonal profiles and physical examination were performed on 19 males and 16 females ages 16–34 years with PWS. Gonadotropins, sex-steroids, inhibin B (INB) and anti-Mullerian hormone (AMH) were measured. We defined 4 groups according to the relative contribution of central and gonadal dysfunction based on FSH and INB levels: Group A: primary hypogonadism (FSH >15 IU/l and undetectable INB (<10 pg/ml); Group B: central hypogonadism (FSH <0.5 IU/l, INB <10 pg/ml); Group C: partial gonadal & central dysfunction (FSH 1.5–15 IU/l, INB >20 pg/ml); Group D: mild central and severe gonadal dysfunction (FSH 1.5–15 IU/l, INB < 10 pg/ml.

**Results:**

There were 10, 8, 9 and 8 individuals in Groups A-D respectively; significantly more males in group A (9, 4, 4 and 2; *P* = 0.04). Significant differences between the groups were found in mean testosterone (*P* = 0.04), AMH (*P* = 0.003) and pubic hair (*P* = 0.04) in males and mean LH (*P* = 0.003) and breast development (*P* = 0.04) in females. Mean age, height, weight, BMI and the distribution of genetic subtypes were similar within the groups.

**Conclusions:**

Analysis of FSH and inhibin B revealed four distinct phenotypes ranging from primary gonadal to central hypogonadism. Primary gonadal dysfunction was common, while severe gonadotropin deficiency was rare. Longitudinal studies are needed to verify whether the individual phenotypes are consistent.

## Background

Prader-Willi syndrome (PWS), first described in 1956 [1] is a neurodevelopmental disorder caused by the absence of paternal expression of imprinted genes localized in the 15q11-q13 region. PWS is characterized by severe hypotonia and feeding difficulties in infancy, an insatiable appetite leading to severe obesity in childhood, short stature, dysmorphic features and cognitive and behavioral problems [2-4]. Manifestations of hypogonadism in infancy include genital hypoplasia in females and micropenis and/or cryptorchidism in males [2,3]. Delayed and incomplete pubertal development is documented in almost all patients with PWS; however precocious puberty has also been described [5]. Some females undergo spontaneous menarche but most have primary or secondary amenorrhea or oligomenorrhea [6]. Pregnancies have been reported in three women with genetically documented PWS thus demonstrating the variable degrees and expression of hypogonadism in this condition [7,8]. No cases of paternity have been described in PWS males.

Hypogonadism in PWS was generally considered to be of central, hypothalamic origin [2,6,9,10]. We previously reported that primary testicular dysfunction is a major contributor to the hypogonadism seen in males with PWS and that variable combinations of primary ovarian defect and hypothalamic dysfunction contribute to the hypogonadism in PWS women [11,12]. The heterogeneous patterns of reproductive hormones indicate that some PWS women may potentially be fertile, while others may require hormone replacement therapy. Recently, Radicioni et al confirmed the heterogeneity of reproductive hormone profiles in PWS [13].

In view of the physical and psycho-social consequences of hypogonadism in this population, hormonal treatment for affected individuals needs to be considered. There are, however, no clear therapeutic guidelines for treating hypogonadism in this population. The aim of the present study was to characterize the etiology and spectrum of reproductive phenotypes of PWS patients in order to provide a basis for routine evaluation of gonadal function and individual treatment options.

## Methods

The procedure was approved by the local ethical committee. It was explained to patients and informed consent was obtained from parents, guardians or adult patients.

### Patients

Of the 112 (52 females) known PWS individuals in Israel, almost all are treated in the multidisciplinary national referral clinic at Shaare Zedek Medical Center, Jerusalem. In this report, we describe our findings in 35 adolescents and adults (16 females) ages 16–34 years. The study group includes 15 males and 14 females some of whose hormonal data appear in our previous publications (11, 12). An additional 2 patients (aged 20 and 28 years) refused to participate and a 21 year-old man was excluded since he was treated with testosterone replacement at the time of the study. No patient was treated with sex hormones for at least six months prior to the study. One 28 year-old man was treated with testosterone for several months but stopped the treatment due to behavioral side effects 10 years before the study. One 20 year-old woman received progyluton [sequential estradiol valerate 2 mg (11 tabs) and estradiol valerate 2 mg plus norgestrel 0.5 mg (11 tablets) (Schering AG/Berlin, Germany)] for several years until 6 months prior to the study. These two were excluded from analyses of height, BMI and the degree of sexual development.

One woman was treated with l-thyroxine for acquired primary hypothyroidism. Two women with diabetes mellitus were treated with insulin. Other medications included risperdone in 5 women, selective serotonine-reuptake inhibitors (SSRIs) in 5 and topiramate in 2. Three subjects (two men aged 27 and 28 and one woman aged 21 years) received growth hormone several years before the study.

### Clinical assessment and hormonal examinations

Pubertal development was evaluated using the Tanner classification [14,15]. Serum concentrations of LH, FSH, estradiol, testosterone, TSH, and prolactin were measured using DxI 800 (Beckman Coulter Instruments Inc., USA). Assay sensitivities were 0.1 IU/L, 0.1 IU/L, 15 pg/ml, 0.1 ng/ml, 0.03 U/ml, and 0.5 ng/ml, respectively. The estradiol intra-assay coefficients of variation (CV) were 6.3–15% for levels ≥40 pg/ml and 20% for levels <40 pg/ml. Inter-assay and intra-assay CVs were less than 7% for other measurements. Dehydroepiandrosterone sulfate (DHEAS), androstenedione, and SHBG concentrations were measured using immunochemiluminescence on IMMULITE (Siemens, Diagnostic Product Corporation, Los Angeles, CA). Assay sensitivities were 0.1 μg/dl, 0.3 ng/ml, and 0.02 nmol/l, respectively, and inter- and intraassay coefficients of variation were less than 10%.

Serum INB and AMH concentrations were measured using highly sensitive two-site ELISAs (Diagnostic Systems Laboratories, Webster, TX). The assay sensitivities were 7 pg/ml and 0.017 ng/ml, respectively. Inter- and intra-assay coefficients of variation were 15 and 7% for INB and 8.7 and 5.3% for AMH, respectively.

Published data from Esoterix Labs (Austin, TX) were used as normal reference ranges of data for hormone levels in adolescents and young adults except for AMH, INB, and SHBG [16]. Appropriate control data for AMH, INB, and SHBG were obtained from published reference data [17-21].

We utilized FSH and INB levels to define 4 groups according to the relative contributions of central and gonadal dysfunction: Group A: primary hypogonadism (FSH >15 IU/l and undetectable (<10 pg/ml) INB; Group B: central hypogonadism (FSH <0.5 IU/l, INB <10 pg/ml); Group C: partial gonadal and central function (FSH 1.5–15 IU/l, INB >20 pg/ml); Group D: mild central and severe gonadal dysfunction (FSH 1.5–15 IU/l, INB < 20 pg/ml. In the last 2 groups FSH levels were within the normal range but were inappropriately low for the low INB.

### Statistical analysis

One-way ANOVA was performed to analyze intergroup differences in the levels of hormone levels and markers of sexual maturation; Bonferroni correction was used as a post-hoc test. Multiple t-tests, Chi-square and Fisher Exact tests were used to analyze gender differences in hormonal levels, the genetic subtypes and the male/female ratio in the different groups. Pearson’s correlations were calculated between the different parameters. *P* < 0.05 was considered as statistically significant.

## Results

As shown in Table 1, 10 patients (9 males) met the established criteria for primary (hypergonadotrophic) hypogonadism (group A) and 8 (4 males) for secondary (central, hypogonadotrophic) hypogonadism (group B). There were 9 individuals in Group C (4 males) and 8 in group D (2 males). The difference in the sex ratios was statistically significant (*P* = 0.04, χ2 test).

**Table 1:** Demographic and clinical data on the 35 participants with PWS: Group A: primary hypogonadism; Group B: central hypogonadism; Group C: partial gonadal & central dysfunction; Group D: mild central and severe gonadal dysfunction

	***Group A (n=10)***	***Group B***^***2***^***(n=8)***	***Group C (n=9)***	***Group D***^***3***^***(n=8)***
Males/Females^4^	9 / 1	4/ 4	4 / 5	2 / 6
Genetic subtype DEL/UPD/IC	8 / 2	5 / 2 / 1	4 / 5	4 / 4
Age (y) (mean±SD)				
Males	23.7±6.3	21.2±5.5	21.5±4.9	19.5±2.1
Females	17.4	24.0±2.7	24.2±4.9	23.2±5.7
Height (cm)				
Males	154.0±8.2	152.0±5.3	155.6±9.5	151.5±4.2
Females	154	142.5±11.6	148.3±4.1	141.1±13.0
BMI (cm/kg^2^)				
Males	26.2±3.8	36.6±4.7	35.3±17.2	28.4±8.1
Females	28.7	35.5±1.6	37.2±15.2	36.7±13.3
Pubic hair^1^				
Males	4.2±0.8	2.3±1.5	3.8±1.3	4.5±0.7
Females	3	4.0±0.0	4.6±0.5	3.0±1.7
Breast development ^1^	3	4.0±0.0	4.4±0.6	3.4±0.5
Penis length (cm)	8.2±1.7	5.3±0.3	8.5±3.7	7.3±1.8
Testes volume ^1^	1.7±0.9	1.5±1.0	2.6±1.3	1.5±0.7

^1^ Tanner stages (13, 14).

^2^ One man who was treated with testosterone for few months about 10 years before the study was excluded from the morphometric and sexual development analysis. His penile length was 8 cm, PH Tanner stage 4 and testes Tanner stage 1.

^3^ One woman who has been treated with sequential estradiol and progestin (progyluton) for a few years before the study was excluded from the morphometric and sexual development analysis. She had breast and PH Tanner stage 5.

^4^*P* = 0.04 for the difference in the sex ration, χ2 test.

**Figure 1:**
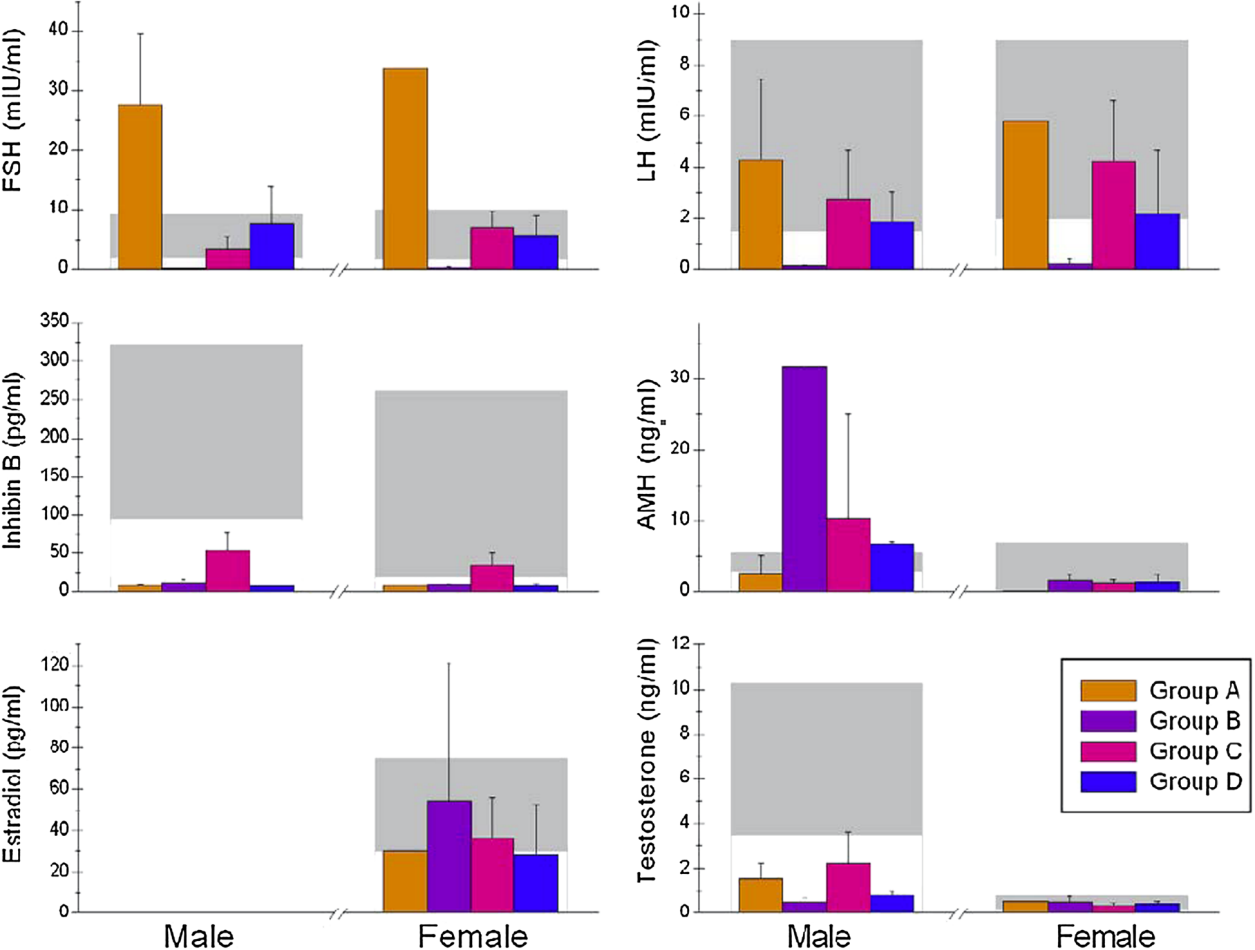
**Serum levels of LH, FSH, AMH and inhibin B (INB) in the four groups of PWS males and females, serum estradiol levels in females and testosterone levels in males.** Group A: primary hypogonadism; Group B: central hypogonadism; Group C: partial gonadal & central dysfunction; Group D: mild central and severe gonadal dysfunction. Criteria for classifying patients in each group according to inhibin B and FSH levels are described in “Results.” The grey bars represent the normal ranges. Among the 4 groups *P* < 0.0001 for the differences in FSH and INB, *P* < 0.001 for the differences in LH and *P* < 0.01 for AMH. Among the males, *P* < 0.0001, *P* < 0.03 and *P* < 0.01 respectively for INB, testosterone and AMH. Among the females, *P* < 0.001 for INB (ANOVA).

Analysis of FSH and INB levels in the groups we defined, revealed highly significant differences among the 4 groups for both males (*P* < 0.0001, ANOVA), and females (*P* < 0.0001 and *P* < 0.001 for FSH and INB respectively) (Figure 1), as expected for the study design. Similar differences in LH levels were found between the groups (*P* < 0.001), however, the levels were within the normal range in most individuals except for those in group B who had very low levels of both FSH and LH.

INB levels were well below the normal range in the males from each of the 4 groups, although the men in group C had significantly higher levels, as per the study design. Only the women in group C and one 17 year-old boy had INB levels approaching the lower limit of the normal range.

Elevated LH and/or FSH levels, indicative of appropriate hypothalamic-pituitary response to hypogonadism (hypergonadotrophic hypogonadism), were found in 9/19 (47%) of the males but in only one female (6%). In addition, INB was below the fifth percentile for 18/19 males and below the 10^th^ percentile in all males (18); in females, INB levels were only moderately decreased (20) (Figure 1). One male and one female in group B and one male in group C had received growth hormone treatment earlier in childhood.

Significant differences were also found among the males groups in testosterone and AMH levels, but only AMH showed significant differences after Bonferroni correction (P = 0.003). Similar to INB, testosterone levels were below the normal range in all males; yet mean testosterone levels in group C were higher than in the other groups. AMH levels in males from group B were in the range of pre- or early pubertal boys which are considerably higher than during late puberty and adulthood in normal males [22,23]. Interestingly, the males in the other groups had almost normal AMH despite very low INB. In contrast, almost all females had AMH levels within the normal range (Figure 1).

Mean age, the genetic diagnoses, height, weight and BMI, as well as pubertal staging were similar within the groups, except for breast development in the females (P = 0.04) and pubic hair Tanner stage in the males (P = 0.04); however the differences did not reach statistical significance after Bonferroni correction. In all males penile length was near or below −2 SD [24,25].

In contrast to testosterone levels in the males, E_2_ levels in the females were within the normal early follicular stage range in most women from all 4 groups. Mean androstenedione, DHEAS, SHBG, prolactin and TSH levels were similar among the four groups.

No correlation was found between the various markers of pubertal development (breast and pubic hair Tanner stages) and any hormone level in females; however, in males, penis length correlated significantly with testosterone levels (r = 0.625; *P* = 0.004) and with testes Tanner stage (r = 0.689; *P* = 0.001) (Table 2).

**Table 2:** **Significant Pearson’s coefficient correlations(r >** 0.550; ***P*** **< 0.02) between the various clinical, hormonal and sexual maturation parameters**

	***Males***		***Females***	
	**r**	**p**	**r**	**p**
LH-FSH	0.636	0.003	0.835	0.000
E2 - androstendione			0.623	0.01
E2 - T			0.639	0.008
T - penis length	0.625	0.004		
E2 - DHEAS			0.757	0.000
DHEAS - androstenedione			0.757	0.000
T - androstenedione			0.853	0.000
T - DHEAS			0.883	0.000
SHBG - INB			0.693	0.003
BMI – penis length	−0.690	0.001		
BMI - PH	−0.594	0.007		
PH – penis length	0.635	0.003		
Testes – penis length	0.663	0.002		
Age - SHBG	0.785	0.000		
E2 - height			−0.598	0.014

Significant correlation was found between estradiol and androgens levels [androstenedione (r = 0.623; *P* < 0.01); testosterone (r = 0.639; *P* < 0.01); DHEA-S (r = 0.757; *P* < 0.0001)], LH and FSH levels (r = 0.636; *P* < 0.003 in males and r = 0.757 in females; *P* < 0.0001) and between androgen levels in females (r = 0.757-0.883; *P* < 0.0001, Table 2).

No correlation was found between the genetic subtype and the degree of sexual maturation or between weight, BMI or Tanner stage in both sexes. There was also no correlation between pubic hair and breast or penis Tanner stages or between age and sexual maturation.

## Discussion

In a cohort of adolescents and adults with PWS, we characterized four distinct patterns of gonadal dysfunction based on FSH and inhibin B levels: hypergonadotrophic (primary gonadal) hypogonadism, hypogonadotrophic (central/hypothalamic) hypogonadism, partial gonadal and central dysfunction and mild central with severe gonadal dysfunction. All our PWS males had immature genital development and low serum testosterone. All of the women had amenorrhea or oligomenorhea although serum estradiol levels were in the normal early follicular range. Although significant differences among the four groups were observed for LH and in men, for testosterone and AMH, we found FSH and INB basal levels to be the most useful criteria for characterizing the type of hypogonadism in our male and female PWS patients.

Reviews of PWS published as recently as 2011 maintain the commonly held dogma that hypogonadism in PWS is due to hypothalamic dysfunction (2, 10). In contrast, we found that severe gonadal dysfunction was the major cause of hypogonadism in 51% of our study population (groups A + D), while severe, isolated gonadotropin deficiency was found in only 23% (group B). These phenotypes showed no correlation with genotype, age or BMI. Gonadal dysfunction, as indicated by INB levels (gender specific percentiles) was less severe in females. These gender differences may explain the potential for fertility in women with PWS [7,8] compared to the apparent lack of fertility in all PWS men.

Hypogonadism in PWS is almost universal, although clinical manifestations are variable [26]. Spontaneous menstruation does occur in some PWS women and rare pregnancies have been reported. In a population-based study Butler et al [27] reported that 8 of 16 females, over 11 years of age had a history of at least one instance of spontaneous vaginal bleeding, without prior administration of sex hormones. Menarche occurred late (at age of 20 ± 7 years) [27]. In our group spontaneous menarche occurred only in 25% of the women (4/16) at age15–30 years. Vaginal bleeding in obese women does not necessarily indicate ovulation or ovarian steroid secretion, since aromatization of adrenal steroids to estrogens in adipose tissue may trigger pseudo-pubertal development, endometrial estrogenisation and breakthrough bleeding. The significant correlation between estradiol and androgens levels found in our females with PWS, including the correlation with the exclusively adrenal steroid DHEA-S, supports this concept.

Five pregnancies have been reported in three women with genetically documented PWS [7,8]. Seven more pregnancies in two women in whom PWS was diagnosed by clinical criteria only, were reported before the genetics of PWS had been delineated [28,29]. There are, however, no known physical characteristics or hormonal patterns which have been shown to predict which PWS are potentially fertile. In two women in our study, ages 21.5 and 26 years, INB levels, 47.3 and 53 pg/ml were consistent with a possibility of fertility [20,30]. We suggest measuring INB in all adolescents and women with PWS, to assess fertility potential and to consider contraception when appropriate.

In normal women, both INB and AMH can be used to assess fertility potential, although AMH is considered to be more reliable [21,30,31]. However, despite the highly significant differences in INB levels between our groups, no inter-group differences could be found in AMH levels in the females (Figure 1). In our previous publications, we showed that both males and females with PWS had a unique pattern of gonadal dysfunction characterized by extremely low or undetected INB along with normal AMH levels [11,12,32]. Thus, in PWS, INB appears to be the most reliable marker of gonadal function and probably of fertility potential.

Hypogonadotrophic hypogonadism was more profound in females: adequate hypothalamic response to the primary gonadal defect (hypergonadotrophic hypogonadism) was found in only one female (6%) in our study, but was documented in 47% of the males. Eiholzer et al [33,34] showed that primary testicular dysfunction contributes to the hypogonadism in PWS male infants and boys. Radicioni et al [13] characterized the hormonal profiles of 24 pubertal PWS males based on LH, FSH, inhibin B and testosterone to hypogonadotropic hypogonadism of central origin for LH (with low secretion of testosterone) and/or FSH(with low secretion of inhibin B), early primary testicular dysfunction secondary to Sertoli cells damage (elevated FSH and low inhibin B) and a combined form of hypogonadism . They did not present data on PWS females.

The dissimilarity, in hypothalamic-pituitary function, between males and females with PWS might be explained by the divergence in the regulation of FSH secretion between normal males and females. In both sexes, FSH secretion is stimulated by GnRH and inhibited by inhibin and, in women, also by estrogens. The normal range of INB levels is higher in males than females at all ages [18-20,35-37]; estradiol levels in women with normal ovarian function are much higher than in men while FSH levels are similar in both sexes. As we proposed previously [12], we assume that the common cause of hypogonadism in most PWS patients, both males and females, is related to a unique defect in INB secretion. The lack of negative feedback by INB was associated with higher FSH levels in males than in females, in whom estradiol and estrone originates from the abundant adipose tissue, and may contribute to the negative feedback on GnRH and FSH secretion.

Guidelines for hormonal replacement therapy in individuals with PWS may need to be tailored individually depending on the specific hypogonadal phenotype. Assessment of specific hormonal data, including INB, together with consideration of other parameters such as BMI, may enable us to tailor specific HRT for women with PWS. Cyclic progesterone may be indicated for those with amenorrhea and normal estradiol levels, particularly in obese women. Progestin would overcome the unopposed estrogenic effect on the endometrium and will prevent endometrial hyperplasia and cancer. Combined estrogen/progesterone treatment or bi-phosphonates is suggested for women with amenorrhea and low estradiol levels, particularly those with low or normal BMI. These will prevent osteoporosis and other consequences of prolonged hypoestrogenism. The possibility that PWS women might need contraception should be considered and discussed with the patient or her guardians if INB is greater than 20 pg/ml. Such INB level indicates partial ovarian function and raises the possibility of fertility. Since all males have low testosterone levels, testosterone treatment should be considered for those from each of the four groups, although behavioral changes should be monitored. A prospective study of HRT for adolescents and adults with PWS including effects on osteoporosis, body image, behavior, and quality of life is needed.

## Conclusions

Analysis of FSH and INB serum levels allowed us to characterize four distinct phenotypes of hypogonadism in PWS adolescents and adults ranging from primary gonadal to central hypogonadism. Severe primary gonadal dysfunction in combination with a wide range of gonadotropin secretion was the major cause of hypogonadism in most of the cohort. Severe, isolated gonadotropin deficiency was found in less than a quarter of our population. These phenotypes showed no correlation with genotype, age or BMI. Hypergonadotrophic hypogonadism was more pronounced in males. Hormonal replacement therapy should be tailored to the specific phenotype. Measurement of INB is recommended for assessing the possibility of fertility in women and the need for contraception. Further longitudinal studies are needed to document whether the individual hypogonadal phenotype is consistent during their life span.

## Competing interests

The authors declare that they have no competing interests.

## Authors’ contributions

VGT enrolled the subjects, participated in critical discussion and writing the manuscript. HH participated in data collection and analysis and revising the manuscript, FB examined all subjects and in the revision of the manuscript, TEG participated in data collection and analysis, critical discussions and writing of the manuscript. All authors read and approved the final manuscript.

## References

[B1] Prader A Labhart A Willi H Ein Syndrom von Adipositas, Kleinwuchs, Kryptorchismus und Oligophrenic nach Myatonicartigem Zustand im Neugeborenalter Schweiz Med Wochenschr 1956 86 1260 1261

[B2] Cassidy SB Driscoll DJ Prader-Willi syndrome Eur J Hum Genet 2009 17 3 13 10.1038/ejhg.2008.165 18781185 PMC2985966

[B3] Goldstone AP Holland AJ Hauffa BP Hokken-Koelega AC Tauber M Recommendations for the diagnosis and management of Prader-Willi syndrome: J Clin Endocrinol Metab 2008 93 4183 4197 10.1210/jc.2008-0649 18697869

[B4] Benarroch F Hirsch HJ Genstil L Landau Y Gross-Tsur V Prader-Willi syndrome: medical prevention and behavioral challenges Child Adolescent Psychiatr Clin N Am 2007 16 695 708 10.1016/j.chc.2007.03.007 17562587

[B5] Pusz ER Rotenstein D Treatment of precocious puberty in a female with Prader-Willi syndrome J Pediatr Endocrinol Metab 2008 21 495 500 18655533 10.1515/JPEM.2008.21.5.495

[B6] Crinò A Schiaffini R Ciampalini P Spera S Beccaria L Benzi F Bosio L Corrias A Gargantini L Salvatoni A Tonini G Trifirò G Livieri C Genetic obesity study group of Italian society of pediatric endocrinology and diabetology (SIEDP) Hypogonadism and pubertal development in Prader-Willi syndrome. Eur J Pediatr 2003 162 327 333 10.1007/s00431-002-1132-4 12692714

[B7] Akefeldt A Törnhage CJ Gillberg C A woman with Prader-Willi syndrome gives birth to a healthy baby girl Dev Med Child Neuro 1999 41 789 790 10.1017/s0012162299221573 10576646

[B8] Schulze A Mogensen H Hamborg-Petersen B Graem N Ostergaard JR Brøndum-Nielsen K Fertility in Prader-Willi syndrome: a case report with Angelman syndrome in the offspring Acta Paediatr 2001 90 455 459 11332942

[B9] Burman P Ritze´n EM, Lindgren AC: Endocrine dysfunction in Prader-Willi syndrome: a review with special reference to GH Endocr Rev 2001 22 787 799 10.1210/er.22.6.787 11739333

[B10] McCandless SE Clinical Report Health Supervision for Children with Prader-Willi syndrome Pediatrics 2011 127 195 204 21187304 10.1542/peds.2010-2820

[B11] Hirsch HJ Eldar-Geva T Benarroch F Rubinstein O Gross-Tsur V Primary testicular dysfunction is a major contributor to abnormal pubertal development in males with Prader-Willi syndrome J Clin Endocrinol Metab 2009 94 2262 2268 10.1210/jc.2008-2760 19401370

[B12] Eldar-Geva T Hirsch HJ Benarroch F Rubinstein O Gross-Tsur V Hypogonadism in females with Prader-Willi syndrome from infancy to adulthood: variable combinations of a primary gonadal defect and hypothalamic dysfunction Eur J Endocrinol 2010 162 377 384 10.1530/EJE-09-0901 19946044

[B13] Radicioni A Di Giorgio G Grugni G Cuttini M Losacco V Anzuini A Soera S Marzano C Lenzi A Cappa M Crino A Multiple forms of hypogonadism of central, peripheral or combined origin in males with Prader Willi sundrome Clin Endocrinol (Oxf) 2011 10.1111/j.1365-2265.2011.04161.x 21718342

[B14] Tanner JM Growth at adolescence 1962 2 Blackwell Scientific, Oxford

[B15] Carel JC Leger J Precocious Puberty New Eng J Med 2008 358 2366 2377 10.1056/NEJMcp0800459 18509122

[B16] Endocrinology expected values and SI unit conversion pocket book. Austin, TX: Esoterix Laboratory Services, Inchttp://www.esoterix.com/files/expected values.pdf

[B17] Lee MM Donahoe PK Hasegawa T Silverman B Crist GB Best S Hasegawa Y Noto RA Schoenfeld D MacLaughlin DT Mullerian Inhibiting Substance in humans: normal levels from infancy to adulthood J Clin Endocrinol Metab 1996 81 571 576 10.1210/jc.81.2.571 8636269

[B18] Andersson AM Juul A Petersen JH Müller J Groome NP Skakkebaek NE Serum Inhibin B in healthy pubertal and adolescent boys: relation to age, stage of puberty, and follicle-stimulating hormone, luteinizing hormone, testosterone, and estradiol levels J Clin Endocrinol Metab 1997 82 3976 3981 10.1210/jc.82.12.3976 9398699

[B19] Jensen TK Andersson AM Jørgensen N Andersen AG Carlsen E Petersen JH Skakkebaek NE Body mass index in relation to semen quality and reproductive hormones among 1,558 Danish men Fertil Steril 2004 82 863 870 10.1016/j.fertnstert.2004.03.056 15482761

[B20] Sehested A Juul AA Andersson AM Petersen JH Jensen TK Muller J Skakkebaek NE Serum inhibin A and inhibin B in healthy prepubertal, pubertal, and adolescent girls and adult women: relation to age, stage of puberty, menstrual cycle, follicle-stimulating hormone, luteinizing hormone, and estradiol levels J Clin Endocrinol Metab 2000 85 1634 1640 10.1210/jc.85.4.1634 10770209

[B21] Sørensen K Andersson AM Skakkebæk NE Juul A Serum sex hormone-binding globulin levels in healthy children and girls with precocious puberty before and during gonadotropin-releasing hormone agonist treatment J Clin Endocrinol Metab 2007 92 3189 3196 10.1210/jc.2007-0231 17519314

[B22] Aksglaede L Sørensen K Boas M Mouritsen A Hagen CP Jensen RB Petersen JH Linneberg A Andersson AM Main KM Skakkebæk NE Juul A Changes in anti-Müllerian hormone (AMH) throughout the life span: a population-based study of 1027 healthy males from birth (cord blood) to the age of 69 years J Clin Endocrinol Metab 2010 95 5357 5364 10.1210/jc.2010-1207 20843948

[B23] Rey R Lordereau-Richard I Carel JC Barbet P Cate RL Roger M Chaussain JL Josso N Anti-Mullerian hormone and testosterone serum levels are inversely related during normal and precocious pubertal development J Clin Endocrinol Metab 1993 77 1220 1226 10.1210/jc.77.5.1220 8077315

[B24] Schonfeld WA Beebe GW Normal growth and variation in the male genitalia from birth to maturity J Urol 1942 48 759 777

[B25] Keefer JR Siberry GK, Iannone R Endocrinology Harriet Lane Handbook 2000 MO: Mosby, St. Louis 277

[B26] Diene G Mimoun E Feigerlova E Caula S Molinas C Grandjean H Tauber M French Reference Centre for PWS: Endocrine disorders in children with Prader-Willi syndrome–data from 142 children of the French database Horm Res Pædiatr 2010 74 121 128 10.1159/000313377 20395666

[B27] Butler JV Whittington JE Holland AJ Boer H Clarke D Webb T Prevalence of, and risk factors for, physical ill-health in people with Prader-Willi syndrome: a population-based study Dev Med Child Neurol 2002 44 248 255 10.1017/S001216220100202X 11995893

[B28] Hockey A Byrne G Cohen A Precocious puberty in the male offspring of a mother and daughter with the Prader-Willi syndrome Am J Med Genet 1987 26 749 10.1002/ajmg.1320260333 3565489

[B29] Laxova R Gilderdale S Ridler MAC An aetiolgical study of fifty-three female patients from a subnormality hospital and of their offspring J Ment Defic Res 1973 17 193 225 4211793 10.1111/j.1365-2788.1973.tb01204.x

[B30] Kwee J Schats R McDonnell J Themmen A de Jong F Lambalk C Evaluation of anti- Mullerian hormone as a test for the prediction of ovarian reserve Fertil Steril 2008 90 737 743 10.1016/j.fertnstert.2007.07.1293 17923131

[B31] Ledger WL Clinical utility of measurement of anti-mullerian hormone in reproductive endocrinology J Clin Endocrinol Metab 2010 95 5144 5154 10.1210/jc.2010-0701 21131535

[B32] Eldar-Geva T Hirsch HJ Rabinowitz R Benarroch F Rubinstein O Gross-Tsur V Primary ovarian dysfunction contributes to the hypogonadism in Women with Prader-Willi Syndrome Horm Res 2009 72 153 159 10.1159/000232489 19729946

[B33] Eiholzer U l’Allemand D Rousson V Schlumpf M Gasser T Girard J Grüters A Simoni M Hypothalamic and gonadal components of hypogonadism in boys with Prader-Labhart- Willi syndrome J Clin Endocrinol Metab 2006 91 892 898 16352691 10.1210/jc.2005-0902

[B34] Eiholzer U Grieser J Schlumpf M l’Allemand D: Clinical effects of treatment for hypogonadism in male adolescents with Prader-Labhart-Willi syndrome Horm Res 2007 68 178 184 17374959 10.1159/000100925

[B35] Crofton PM Evans AEM Groome NP Taylor MRH Holland CV Kelnar CJH Dimeric inhibins in girls from birth to adulthood: relationship with age, pubertal stage, FSH and oestradiol Clin Endocrinol (Oxf) 2002 56 223 230 10.1046/j.0300-0664.2001.01449.x 11874414

[B36] Crofton PM Evans AE Groome NP Taylor MR Holland CV Kelnar CJ Inhibin B in boys from birth to adulthood: relationship with age, pubertal stage, FSH and testosterone Clin Endocrinol (Oxf) 2002 56 215 221 10.1046/j.0300-0664.2001.01448.x 11874413

[B37] Foster CM Olton PR Racine MS Phillips DJ Padmanabhan V Sex differences in FSH-regulatory peptides in pubertal age boys and girls and effects of sex steroid treatment Hum Reprod 2004 19 1668 1676 10.1093/humrep/deh284 15155605

